# Association Between Emotional Intelligence and Work Outcomes Among Midwives in Northern Ghana: A Path Analysis Study

**DOI:** 10.1155/jonm/6200182

**Published:** 2026-10-07

**Authors:** Letitia Chanayireh, Zariatu Yakubu, Sheila Kansig Puotege, Beatrice Asakiya, Mary Tusungu, Mudasir Mohammed Ibrahim, Iddrisu Mohammed Sisala, Abubakari Wuni

**Affiliations:** ^1^ Department of Midwifery and Women’s Health School of Nursing and Midwifery University for Development Studies Tamale Ghana uds.edu.gh; ^2^ Department of Obstetrics and Gynecology Tamale Teaching Hospital Tamale Ghana tamaleteachinghospital.org; ^3^ Department of Internal Medicine (M3) Tamale Teaching Hospital Tamale Ghana tamaleteachinghospital.org; ^4^ Department of Nursing Nurses’ and Midwives’ Training College Tamale Ghana; ^5^ College of Nursing University of Kentucky Lexington Kentucky USA uky.edu

**Keywords:** emotional intelligence, job performance, midwives, organizational commitment, work engagement

## Abstract

**Background:**

Emotional intelligence (EI) is increasingly recognized as an important factor influencing work outcomes. However, evidence on EI among midwives, particularly in northern Ghana, remains limited.

**Objective:**

This study assessed the association between self‐reported EI and organizational commitment, work engagement, and job performance among midwives in the Tamale Metropolis.

**Methods:**

A quantitative cross‐sectional analytical design was employed among midwives (*N* = 254) working in the Tamale Metropolis. Data were analyzed using SAS JMP Pro Version 17.1 and R software Version 4.2.3.

**Results:**

Overall, only 13.4% of the midwives demonstrated high EI, indicating that relatively few participants reported high levels of this important professional competency. Path analysis revealed that EI was positively associated with organizational commitment (*β* = 0.34, 95% confidence interval (CI): [0.21, 0.47], *p* < 0.001), work engagement (*β* = 0.37, 95% CI: [0.27, 0.45], *p* < 0.001), and job performance (*β* = 0.41, 95% CI: [0.31, 0.51], *p* < 0.001). EI was also positively associated with normative commitment (*β* = 0.33, 95% CI: [0.22, 0.44], *p* < 0.001), continuance commitment (*β* = 0.26, 95% CI: [0.15, 0.37], *p* < 0.001), affective commitment (*β* = 0.20, 95% CI: [0.04, 0.36], *p* = 0.013), absorption (*β* = 0.34, 95% CI: [0.24, 0.44], *p* < 0.001), dedication (*β* = 0.36, 95% CI: [0.27, 0.45], *p* < 0.001), vigor (*β* = 0.25, 95% CI: [0.15, 0.35], *p* < 0.001), task performance (*β* = 0.38, 95% CI: [0.29, 0.47], *p* < 0.001), and contextual performance (*β* = 0.42, 95% CI: [0.33, 0.53], *p* < 0.001). Age was the only significant predictor in adjusted analysis, limiting explanatory power.

**Conclusions:**

EI was positively associated with organizational commitment, work engagement, and job performance among midwives. Conclusions are based on a single metropolis, cross sectional design limiting causal attribution. However, incorporating EI training into midwifery education and professional development may strengthen workforce effectiveness.

**Implications for Nursing Management:**

EI may be a relevant consideration in midwifery workforce development and professional development initiatives. However, such initiatives should complement broader organizational measures addressing working conditions and other factors that influence midwives’ workplace outcomes.

**Patient or Public Contribution:**

No patient or public contribution.

## 1. Introduction

Midwives’ experiences of frontline healthcare delivery in rural maternal and newborn care have received limited scholarly attention over the past 2 decades in low‐ and middle‐income countries (LMICs), particularly in resource‐constrained settings [1]. Midwives in these contexts encounter numerous challenges, including psychological and emotional distress, inadequate professional support, insufficient infrastructure, workforce shortages, financial constraints, and weak or ineffective health policies. These challenges can undermine their capacity to deliver high‐quality maternal and newborn care [2, 3].

In Ghana, including the Northern Region, midwives play a critical role in providing emergency obstetric and neonatal care, often serving as primary skilled healthcare providers in rural and periurban settings where doctors are scarce [4, 5]. Despite their essential role, midwives continue to face significant occupational challenges, including excessive workloads, limited professional autonomy, inadequate resources, poor motivation, and chronic understaffing [2]. These systemic and structural challenges may be compounded by psychological stressors, including persistent pressure, anxiety, demotivation, and emotional exhaustion, which can affect midwives’ well‐being and professional functioning [4, 6]. Following adverse maternal outcomes, such as maternal deaths, midwives may also experience helplessness, grief, and emotional distress, yet limited attention has been given to their psychological well‐being [7, 8].

Emotional intelligence (EI) may be particularly relevant in helping midwives navigate these occupational demands. EI refers to the ability to perceive, understand, regulate, and manage emotions in oneself and in interactions with others, thereby facilitating effective interpersonal relationships and adaptive responses to stress and occupational demands [9]. Research has shown that higher EI is associated with greater work engagement, improved job performance, and stronger organizational commitment among healthcare professionals [10–13]. In Ghana, however, empirical evidence concerning EI among midwives remains limited, and few studies distinguish midwives from nurses despite the distinctive emotional, interpersonal, and professional demands of midwifery practice [14].

Given the structural, systemic, and psychological challenges experienced by midwives in Ghana, particularly in the Tamale Metropolis, understanding the role of EI in their work‐related outcomes is important. Such evidence may inform approaches to strengthening midwives’ emotional competencies, workforce effectiveness, and quality of care. Therefore, the present study assessed the association between self‐reported EI and organizational commitment, work engagement, and job performance among midwives in the Tamale Metropolis.

## 2. Literature Review

### 2.1. Relationship Between EI and Organizational Commitment

Organizational commitment refers to an individual’s emotional attachment, identification, and loyalty to an organization [15]. An earlier study by Abraham [16] demonstrated that individuals with higher levels of EI exhibited greater organizational loyalty [17].

Subsequent research has continued to report a positive relationship between EI and organizational commitment, particularly among nursing professionals. This relationship has also been linked to greater job satisfaction [18–21]. Studies among nurses have further indicated that stronger organizational commitment is associated with greater morale, retention, and positive work‐related outcomes [22–24]. However, evidence specific to midwives remains limited.

### 2.2. Relationship Between EI and Work Engagement

Work engagement, first conceptualized by Kahn in 1990, refers to a positive, fulfilling work‐related state characterized by individuals’ physical, emotional, and cognitive investment in their work roles [11]. The concept emerged partly from the literature on burnout and represents a shift from focusing primarily on occupational stress toward promoting employee well‐being [11, 25]. Work engagement is generally characterized by three dimensions: vigor, dedication, and absorption [11, 26]. Vigor refers to high energy levels and resilience in the face of workplace demands [27]. Dedication embodies a strong emotional and cognitive investment in one’s job, often reflected in enthusiasm, inspiration, pride, and a sense of meaningfulness [28]. Absorption involves being fully immersed in work activities, often to the point where time seems to pass quickly and detachment becomes difficult [29].

A growing body of evidence demonstrates a positive relationship between EI and work engagement, particularly among nurses. Emotionally intelligent healthcare professionals may be better equipped to manage emotional demands, sustain motivation, and remain engaged despite workplace challenges [25, 29–31]. Nevertheless, the extent to which these findings apply specifically to midwives in resource‐constrained settings remains insufficiently established.

### 2.3. Relationship Between EI and Job Performance

Job performance in healthcare refers to the extent to which professionals effectively execute job‐related tasks and contribute to quality patient care and service delivery [32]. It is commonly conceptualized as comprising task performance and contextual performance [33]. Task performance encompasses core technical and nontechnical responsibilities, whereas contextual performance includes behaviors that support the organizational environment, such as teamwork and cooperation [33–35].

Higher EI has been associated with better job performance across these dimensions [36, 37]. Studies among nurses suggest that emotionally intelligent professionals may be better able to manage stress, establish positive relationships with patients and colleagues, and maintain effective work performance [36–38]. However, evidence directly examining this relationship among midwives remains limited.

## 3. Current Research Gap

EI is increasingly recognized as an important individual‐level competency in healthcare because it may support emotional regulation, communication, interpersonal relationships, and adaptive responses to demanding work environments [39, 40]. This may be particularly relevant to midwives, whose work involves sustained interpersonal interaction, emotional support for women and families, and decision‐making under complex and sometimes resource‐constrained conditions [41, 42].

Despite growing evidence linking EI with organizational commitment, work engagement, and job performance, several important gaps remain. First, the existing evidence base has predominantly focused on nurses and broader healthcare populations rather than midwives as a distinct professional group [31, 33, 43]. Although nurses and midwives share some workplace characteristics, midwifery involves distinctive clinical, emotional, and interpersonal responsibilities. Consequently, findings from predominantly nursing populations cannot necessarily be assumed to apply to midwives.

Second, there is limited empirical evidence from Ghana and other resource‐constrained settings examining the relationship between EI and multiple work‐related outcomes among midwives. Existing evidence on organizational commitment [18–21], work engagement [25, 29–31], and job performance [36–38] provides a basis for expecting positive associations with EI, but the applicability of these relationships to Ghanaian midwives remains unclear.

Third, much of the literature has examined EI in relation to individual work outcomes without simultaneously examining organizational commitment, work engagement, and job performance within a midwifery population [18, 19, 25, 31, 36, 37]. The present study, therefore, extends existing evidence by examining these three work‐related outcomes within the same professional and geographical context. Importantly, the study focuses on EI as an individual‐level factor and does not seek to assess organizational‐level determinants of work outcomes or emotional regulation as a separate construct. This delineation is important because organizational factors such as staffing, resources, leadership, and institutional support may influence work outcomes but are beyond the specific scope of the present investigation.

The present study is, therefore, largely confirmatory, as the proposed relationships have been reported in previous healthcare research. However, its contribution lies in generating context‐specific evidence among midwives in the Tamale Metropolis, an understudied professional and geographical population. This evidence may help determine whether previously observed relationships between EI and work‐related outcomes are also evident among Ghanaian midwives working within a resource‐constrained healthcare context.

To address these gaps, the present study assessed the association between self‐reported EI and organizational commitment, work engagement, and job performance among midwives in the Tamale Metropolis. Figure 1 presents the conceptual model underpinning the study. The study addressed the following research questions: (1) What is the level of EI among midwives in the Tamale Metropolis? (2) Do midwives’ sociodemographic characteristics influence their EI levels? and (3) Is EI significantly associated with organizational commitment, work engagement, and job performance among midwives in the Tamale Metropolis?

**FIGURE 1:**
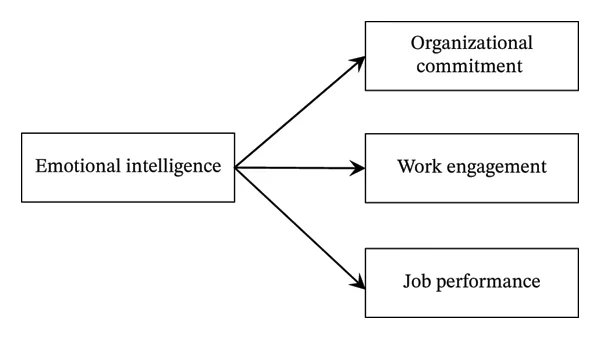
Conceptual model depicting the association between emotional intelligence and organizational commitment, work engagement, and job performance among midwives.

## 4. Methods

### 4.1. Study Design

A quantitative cross‐sectional analytical design was employed to assess the association between self‐reported EI and organizational commitment, work engagement, and job performance among midwives in the Tamale Metropolis. A cross‐sectional design was appropriate because the study aimed to examine associations between EI and work‐related outcomes at a defined point in time rather than evaluate an intervention or establish temporal or causal relationships. A controlled or longitudinal design was, therefore, beyond the scope of the present observational study. This study utilized the same cross‐sectional dataset as that used by Mohammed et al. [14] but addressed a substantively different research question and analytical objective. Mohammed et al. [14] examined the mediating role of work engagement in the relationship between organizational commitment and job performance, whereas the present study examined EI as the primary explanatory variable and its associations with organizational commitment, work engagement, and job performance using path analysis. EI was not examined as a construct in the previous publication. All analyses and interpretations presented in this manuscript are original.

### 4.2. Study Setting and Population

The study was conducted in three major public healthcare institutions in Tamale, the capital of Ghana’s Northern Region: Tamale Teaching Hospital (TTH), Tamale West Hospital (TWH), and Tamale Regional Hospital (TRH). These hospitals operate 24 h a day, providing essential maternal and general healthcare services to residents of northern Ghana and surrounding regions. At the time of the study, the combined workforce included 552 midwives (TTH = 305, TRH = 117, and TWH = 130), forming the target population for this research.

### 4.3. Inclusion and Exclusion Criteria

Midwives were eligible for inclusion if they were formally employed at any of the three hospitals, actively engaged in direct patient care, and provided informed consent to participate in the study. Midwives were excluded if they were assigned solely to administrative roles or were participating in their mandatory national service temporary rotation.

### 4.4. Sample Size Determination and Sampling Method

The required sample size was calculated using Yamane’s [44] formula for finite populations: *n* = *N*/[1 + *N* (*e* ^2^)], where *n* is the sample size, *N* is the total population (552), and *e* is the margin of error (0.05). This yielded a base sample size of 231, which was increased to 254 to account for a 10% nonresponse rate. A stratified sampling strategy was employed, with strata defined by hospital. Stratum sizes were determined using the formula: (stratum population/total population) × sample size. Proportional allocation resulted in the following sample: TTH (305 midwives) – 140 participants, TWH (130 midwives) – 60 participants, and TRH (117 midwives) – 54 participants. Within each stratum, simple random sampling was conducted using ward‐level midwife lists imported into SAS JMP Pro. The software’s random selection function was used to generate the study sample. Of the 254 midwives initially selected, 244 participated, giving an initial response rate of 96.1%. Ten midwives (3.9%) were either unavailable or declined participation and were replaced with randomly selected midwives from the same hospital strata using the same sampling procedure. Following replacement, all 254 targeted participants completed the questionnaire, resulting in a final completion rate of 100%.

### 4.5. Data Collection Instrument

Data were collected using a structured, close‐ended questionnaire comprising five sections. The first section gathered sociodemographic and workplace information, including age, gender, marital status, number of children, education level, years of experience, workplace, professional rank, and prior exposure to EI training. The second section assessed EI using the 33‐item Schutte Self‐Report Emotional Intelligence Test (SSEIT), with responses recorded on a 5‐point Likert scale ranging from 1 (*strongly disagree*) to 5 (*strongly agree*). Three items (5, 28, and 33) were reverse‐coded, and composite scores were calculated by summing item responses across the entire scale. In this study, following the Veritas International Training Center guidelines, total scores above 137 indicated high EI, while scores of 137 or below indicated low EI [45, 46]. The third section measured organizational commitment using the 18‐item Allen and Meyer [47] scale, which includes three subscales: affective commitment (6 items), continuance commitment (6 items), and normative commitment (6 items). Responses were recorded on a 7‐point scale from 1 (*strongly disagree*) to 7 (*strongly agree*), and composite scores were calculated by averaging item responses for the full scale and each subscale, with higher scores reflecting higher commitment.

The fourth section evaluated work engagement using the 9‐item Utrecht Work Engagement Scale (UWES‐9), comprising three subscales—vigor, dedication, and absorption (3 items each)—with responses recorded on a 7‐point Likert scale from 0 (*never*) to 6 (*always*). Average scores were computed for the overall scale and subscales, with higher scores indicating higher engagement [48, 49]. The fifth and final section assessed job performance using the 18‐item Individual Work Performance Questionnaire (IWPQ), covering task performance (5 items), contextual performance (8 items), and counterproductive work behavior (CWB) (5 items). Task and contextual performance items were rated on a 5‐point scale from 0 (*seldom*) to 4 (*always*), while CWB items used a 5‐point scale from 0 (*never*) to 4 (*often*). Average scores were computed for the overall scale and subscales, with higher scores indicating higher performance [50, 51].

### 4.6. Validity and Reliability

The study instrument was administered in English, and all scales had established validity and reliability in prior research. Content and face validity were assessed using the Content Validity Index (CVI) by a panel of six experts who evaluated item relevance. The panel consisted of experts in midwifery practice, nursing/midwifery education, maternal health, and research methodology, with extensive clinical, academic, and research experience relevant to the study topic. All items met the minimum CVI threshold (> 0.80), and the original scale structures were retained without modification. The questionnaire was pilot‐tested with 35 midwives from a nonparticipating hospital to evaluate clarity and feasibility, with no issues identified. Internal consistency in the main study sample was assessed using Cronbach’s alpha. The EI scale demonstrated excellent reliability (*α* = 0.90). Organizational commitment exhibited good overall reliability (*α* = 0.80), with subscale alphas of 0.72 (normative), 0.75 (continuance), and 0.71 (affective). Work engagement showed strong reliability overall (*α* = 0.89), with subscale alphas ranging from 0.71 (vigor) to 0.87 (dedication). Job performance also demonstrated high reliability (*α* = 0.87), with subscale alphas of 0.89 (task performance), 0.80 (contextual performance), and 0.84 (counterproductive behaviors).

### 4.7. Data Collection Procedure

Following ethical and administrative approvals, data were collected from January to March 2025. Written and verbal informed consent was obtained from all participants prior to participation. Questionnaires were administered in person, and participants were allowed to complete them at their own pace under researcher supervision to ensure accuracy. Upon completion, questionnaires were collected, reviewed for completeness, and placed in sealed envelopes labeled with anonymous codes to maintain confidentiality. All completed questionnaires were securely stored to protect participant data.

### 4.8. Data Analysis

Data were analyzed using SAS JMP Pro (Version 17.1) and R (Version 4.2.3). No missing data were identified; therefore, data imputation was not required. Descriptive statistics were computed for continuous variables using means, standard deviations, and 95% confidence intervals (CIs), while categorical variables were summarized using frequencies and percentages. Assumptions for regression analyses were assessed prior to model estimation. Normality of residuals was evaluated using the Shapiro–Wilk test and *Q*–*Q* plots, homoscedasticity was examined using residual‐versus‐fitted plots, and multicollinearity was assessed using variance inflation factors (VIFs). EI violated assumptions of normality and homoscedasticity; therefore, robust regression methods were employed to reduce the influence of outliers and unequal error variance. Bivariate and multivariable robust regression analyses were conducted using the *robustbase* package in R to identify factors associated with EI. All statistical tests were two‐tailed, with statistical significance set at *p* < 0.05.

### 4.9. Path Analysis

Path analysis was conducted to examine the association between EI and organizational commitment, work engagement, and job performance among midwives in the Tamale Metropolis. Observed composite scores for each scale and subscale were used in the analysis. EI was specified as the exogenous variable, while organizational commitment, work engagement, and job performance were modeled as endogenous variables. Path analysis was selected because it enabled the three theoretically related outcomes to be examined simultaneously within a single multivariate framework, while allowing residual correlations to be estimated and maintaining a consistent model structure. This provided a parsimonious representation of the hypothesized associations beyond estimating each outcome in separate linear regression models.

Direct paths from EI to organizational commitment, work engagement, and job performance were specified within a single structural model. Because age could potentially confound these associations, the primary analyses were additionally re‐estimated with adjustment for age as a sensitivity analysis. The estimated associations between EI and each of the three outcomes changed minimally following age adjustment, suggesting that age did not materially confound the observed relationships. This sensitivity analysis therefore provided an assessment of the robustness of the primary findings. However, the age‐adjusted estimates were not presented as additional results in the main manuscript to maintain a parsimonious presentation of the primary model. No covariates were included in the primary path model; thus, the reported path coefficients represent unadjusted associations. Residual correlations among the three endogenous outcomes (organizational commitment, work engagement, and job performance) were freely estimated to account for their shared unexplained variance.

The path model was estimated in R (Version 4.2.3) using the *lavaan* package with maximum likelihood estimation with robust standard errors (MLR) to correct for nonnormality (Supporting File 1). Standardized coefficients were reported to facilitate comparison. The subscale results presented were derived from drawing paths from EI to each subscale within a single structural model, rather than from separate models. Model fit indices were not reported because the specified path model was just‐identified (zero degrees of freedom). Under this condition, global fit indices such as RMSEA, CFI, TLI, and SRMR are necessarily perfect and, therefore, do not provide meaningful information about model adequacy [52]. Interpretation focused on the magnitude, direction, and statistical significance of the estimated associations. All statistical tests were two‐tailed, with statistical significance set at *p* < 0.05.

### 4.10. Ethical Considerations

The study received ethical clearance from the Committee on Human Research, Publications, and Ethics at Kwame Nkrumah University of Science and Technology (Ref: CHRPE/AP/013/24). Permission to conduct the study was also obtained from the respective participating institutions before data collection commenced. All participating midwives were fully informed of the study’s purpose, potential risks, and their rights, including the right to withdraw at any time without consequence. Confidentiality was strictly maintained, and no identifying information was linked to participants’ responses. It should be noted that this study utilized the same dataset as the study by Mohammed et al. [14], involving the same participant cohort. Ethical clearance for both studies was obtained under the same protocol, and the original ethics document and approved study protocol are available upon request.

## 5. Results

### 5.1. Sociodemographic and Workplace Information

A total of 254 midwives participated in the study, achieving full completion among the final recruited sample. The largest proportion of participants (54.3%) were aged 30–39 years. The majority were female (96.9%) and married (72.0%), while 37.0% reported having one or two children. Most participants (59.4%) held a Bachelor’s degree in midwifery. Regarding professional experience, just over half (50.4%) had worked for fewer than 5 years, and staff midwives constituted the largest professional category (46.5%). Although a substantial proportion (75.2%) was familiar with the concept of EI, only 27.6% reported having received formal training on EI (Table 1).

**TABLE 1:** Sociodemographic and workplace information of the midwives (*N* = 254).

Variable	Category	Frequency (*n*)	Percentage (%)
Age in years	20–29	72	28.4
	30–39	138	54.3
	40–49	44	17.3

Gender	Female	246	96.9
	Male	8	3.1

Marital status	Single	71	28.0
	Married	183	72.0

Number of children	None	75	29.5
	1–2	94	37.0
	3–4	81	31.9
	5 or more	4	1.6

Educational level	Diploma in midwifery	98	38.6
	Bachelor’s degree in midwifery	151	59.4
	Master’s degree in midwifery	5	2.0

Work experience	Less than 5 years	128	50.4
	5–10 years	109	42.9
	More than 10 years	17	6.7

Workplace	TTH	140	55.1
	TRH	54	21.3
	TWH	60	23.6

Professional rank	Staff midwife	118	46.5
	Senior staff midwife	115	45.3
	Principal midwifery officer	21	8.3

Have ever heard of emotional intelligence	Yes	191	75.2
	No	63	24.8

Have ever received training on emotional intelligence	Yes	70	27.6
	No	184	72.4

Source: Field data (2025).

### 5.2. Descriptive Statistics of Main Study Variables

The mean score for EI among the participants was 123.00 (SD = 15.00). Organizational commitment had a mean score of 3.05 (SD = 0.54), while the average work engagement score was 2.41 (SD = 0.96). The mean score for job performance was 2.01, with a standard deviation of 0.67 (Table 2).

**TABLE 2:** Descriptive statistics of emotional intelligence, organizational commitment, work engagement, and job performance among midwives (*N* = 254).

Variable	Mean	95% CI (lower)	95% CI (upper)	Standard deviation	Minimum	Maximum
Emotional intelligence	123.00	121.00	125.00	15.00	44.00	153.00
Job performance	2.01	1.93	2.09	0.67	0	4.00
Organizational commitment	3.05	2.98	3.12	0.54	1.17	4.44
Work engagement	2.41	2.29	2.53	0.96	0	4.00

Source: Field data (2025).

### 5.3. Level of EI

Among the participants, only 13.4% were classified as having high EI (95% CI: 10%–18%), as illustrated in Figure 2. Regarding specific emotional competencies, 61.8% reported that they could easily identify their emotions as they arose, and 61.4% indicated awareness of their emotional states during experiences. Additionally, 64.6% stated that they often help others feel better when they are feeling low, while 60.2% reported relying on positive moods to maintain motivation when facing challenges. Furthermore, 64.2% indicated that they draw upon memories of past successes when encountering new difficulties. In contrast, awareness of nonverbal communication was relatively lower, with only 48.0% and 49.6% acknowledging understanding of the nonverbal cues they send and receive, respectively (Table 3).

**FIGURE 2:**
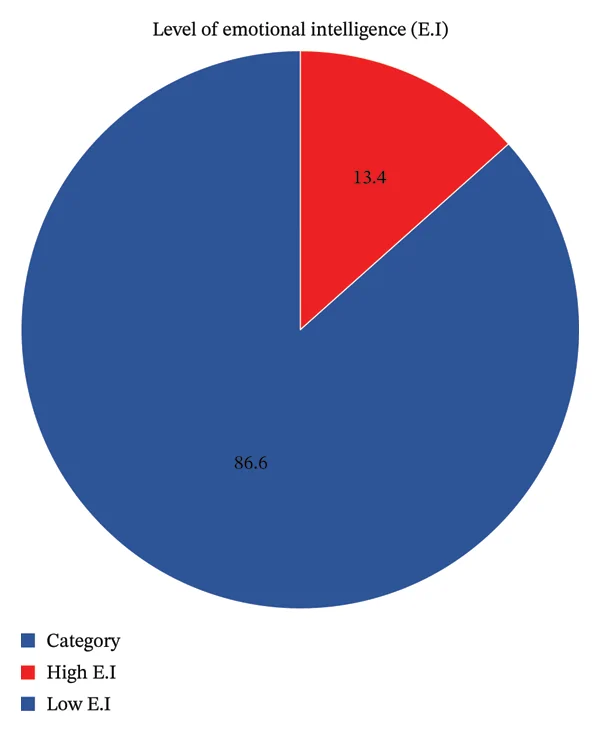
Level of emotional intelligence among midwives (*N* = 254). Source: Field data (2025).

**TABLE 3:** Emotional intelligence among midwives (*N* = 254).

Variable	Strongly disagree		Disagree		Neither disagree nor agree		Agree		Strongly agree	
	*N*	%	*N*	%	*N*	%	*N*	%	*N*	%
1. I know when to speak about my problems to others	14	5.5	7	2.8	20	7.9	131	51.6	82	32.3
2. When I am faced with obstacles, I remember times I faced similar obstacles and overcame them	7	2.8	9	3.5	22	8.7	163	64.2	53	20.9
3. I expect that I will do well in most things I try	10	3.9	10	3.9	26	10.2	151	59.4	57	22.4
4. Other people find it easy to confide in me	13	5.1	22	8.7	48	18.9	126	49.6	45	17.7
5. I find it hard to understand the nonverbal messages of other people	21	8.3	95	37.4	48	18.9	73	28.7	17	6.7
6. Some of the major events of my life have led me to re‐evaluate what is important and not important	6	2.4	15	5.9	20	7.9	142	55.9	71	28.0
7. When my mood changes, I see new possibilities	10	3.9	33	13.0	59	23.2	123	48.4	29	11.4
8. Emotions are one of the things that make my life worth living	6	2.4	31	12.2	48	18.9	125	49.2	44	17.3
9. I am aware of my emotions as I experience them	2	0.8	12	4.7	34	13.4	156	61.4	50	19.7
10. I expect good things to happen	4	1.6	18	7.1	41	16.1	123	48.4	68	26.8
11. I like to share my emotions with others	10	3.9	49	19.3	60	23.6	109	42.9	26	10.2
12. When I experience a positive emotion, I know how to make it last	5	2.0	18	7.1	38	15.0	142	55.9	51	20.1
13. I arrange events others enjoy	4	1.6	28	11.0	86	33.9	113	44.5	23	9.1
14. I seek out activities that make me happy	3	1.2	14	5.5	35	13.8	136	53.5	66	26.0
15. I am aware of the nonverbal messages I send to others	7	2.8	26	10.2	56	22.0	122	48.0	43	16.9
16. I present myself in a way that makes a good impression on others	8	3.1	11	4.3	33	13.0	136	53.5	66	26.0
17. When I am in a positive mood, solving problems is easy for me	5	2.0	8	3.1	27	10.6	117	46.1	97	38.2
18. By looking at their facial expressions, I recognize the emotions people are experiencing	6	2.4	23	9.1	41	16.1	128	50.4	56	22.0
19. I know why my emotions change	8	3.1	21	8.3	55	21.7	131	51.6	39	15.4
20. When I am in a positive mood, I am able to come up with new ideas	6	2.4	11	4.3	29	11.4	127	50.0	81	31.9
21. I have control over my emotions	8	3.1	22	8.7	46	18.1	141	55.5	37	14.6
22. I easily recognize my emotions as I experience them	1	0.4	10	3.9	28	11.0	157	61.8	58	22.8
23. I motivate myself by imagining a good outcome to tasks I take on	3	1.2	9	3.5	27	10.6	141	55.5	74	29.1
24. I compliment others when they have done something well	3	1.2	4	1.6	21	8.3	112	44.1	114	44.9
25. I am aware of the nonverbal messages other people send	5	2.0	20	7.9	64	25.2	126	49.6	39	15.4
26. When another person tells me about an important event in his or her life, I almost feel as though I have experienced this event myself	4	1.6	20	7.9	48	18.9	138	54.3	44	17.3
27. When I feel a change in emotions, I tend to come up with new ideas	7	2.8	34	13.4	57	22.4	119	46.9	37	14.6
28. When I am faced with a challenge, I give up because I believe I will fail	61	24.0	92	36.2	36	14.2	55	21.7	10	3.9
29. I know what other people are feeling just by looking at them	14	5.5	58	22.8	77	30.3	86	33.9	19	7.5
30. I help other people feel better when they are down	4	1.6	5	2.0	26	10.2	164	64.6	55	21.7
31. I use good moods to help myself keep trying in the face of obstacles	3	1.2	5	2.0	27	10.6	153	60.2	66	26.0
32. I can tell how people are feeling by listening to the tone of their voice	6	2.4	13	5.1	42	16.5	145	57.1	48	18.9
33. It is difficult for me to understand why people feel the way they do	21	8.3	88	34.6	66	26.0	60	23.6	19	7.5

Source: Field data (2025).

### 5.4. Factors Associated With EI

Robust regression analysis was conducted to examine factors associated with EI among the participants. At the bivariate level, age and number of children were independently associated with EI. Participants aged 40–49 years demonstrated significantly higher EI compared with those aged 20–29 years (*β* = 7.41, 95% CI: 2.86–11.97, *p* = 0.001). Also, participants with three to four children exhibited higher EI than those with no children (*β* = 3.92, 95% CI: 0.26–7.58, *p* = 0.037). At the multivariate level, age remained the only significant predictor. Participants aged 30–39 years (*β* = 4.57, 95% CI: 0.24–8.89, *p* = 0.039) and 40–49 years (*β* = 9.92, 95% CI: 4.26–15.58, *p* = 0.001) had significantly higher EI compared with their counterparts aged 20–29 years (Table 4).

**TABLE 4:** Robust regression analysis of the association between emotional intelligence and sociodemographic/work characteristics among midwives (*N* = 254).

Variable	Category	Bivariate		Multivariate	
		*β* [95% CI]	*p* value	*β* [95% CI]	*p* value
Age	20–29 years				
	30–39 years	2.39 [−1.00–5.78]	0.170	4.57 [0.24–8.89]	0.039^∗^
	40–49 years	7.41 [2.86–11.97]	0.001^∗^	9.92 [4.26–15.58]	0.001^∗^

Gender	Male				
	Female	−3.48 [−7.54–0.59]	0.100	−3.66 [−8.53–1.21]	0.142

Marital status	Single				
	Married	1.54 [−1.71–4.80]	0.351	−1.21 [−5.30–2.88]	0.562

Number of children	None				
	1‐2	1.26 [−2.29–4.80]	0.487	0.92 [−3.28–5.11]	0.668
	3‐4	3.92 [0.26–7.58]	0.037^∗^	3.31 [−1.20–7.83]	0.152
	5 or more	8.59 [−8.95–26.14]	0.338	3.37 [−13.32–20.07]	0.692

Educational level^†^	Bachelor’s degree in midwifery or higher				
	Diploma in midwifery	−0.21 [−3.41–2.99]	0.898	0.54 [−3.31–4.39]	0.782

Work experience	Less than 5 years				
	5–10 years	0.84 [−2.42–4.09]	0.616	−1.95 [−5.96–2.07]	0.343
	More than 10 years	5.34 [−0.96–11.64]	0.098	0.85 [−6.49–8.19]	0.820

Workplace	TTH				
	TRH	−1.92 [−6.82–2.99]	0.445	−1.22 [−5.97–3.53]	0.614
	TWH	−0.25 [−3.91–3.40]	0.891	−1.40 [−5.55–2.75]	0.507

Professional rank	Principal midwife				
	Senior midwife	−4.36 [−10.01–1.29]	0.132	−2.15 [−9.25–4.95]	0.553
	Staff midwife	−2.29 [−8.04–3.45]	0.435	2.34 [–4.97–9.66]	0.530

Have ever received training on emotional intelligence	No				
	Yes	0.45 [−2.65–3.55]	0.775	1.01 [−2.07–4.10]	0.520

*Note:* ^∗^Statistically significant at *p* < 0.05.

### 5.5. Association Between EI and Organizational Commitment, Work Engagement, and Job Performance

To assess the potential for common method bias, Harman’s single‐factor test was conducted. The first unrotated factor accounted for 20.76% of the total variance, which was below the commonly cited 50% threshold. This finding provides some reassurance that a single dominant factor did not account for most of the variance in the measured variables although Harman’s single factor test alone cannot rule out common method bias. Path analysis was then performed to examine the association between EI and organizational commitment, work engagement, and job performance among the participants.

EI was positively associated with overall organizational commitment (*β* = 0.34, 95% CI: 0.21–0.47, *p* < 0.001), explaining 12% of the variance (*R* ^2^ = 0.12). This positive association extended to all three dimensions of organizational commitment: normative commitment (*β* = 0.33, 95% CI: 0.22–0.44, *p* < 0.001), continuance commitment (*β* = 0.26, 95% CI: 0.15–0.37, *p* < 0.001), and affective commitment (*β* = 0.20, 95% CI: 0.04–0.36, *p* = 0.013).

Similarly, EI was positively associated with work engagement (*β* = 0.37, 95% CI: 0.27–0.45, *p* < 0.001), accounting for 14% of the variance (*R* ^2^ = 0.14). Positive associations were observed across all dimensions of work engagement, including absorption (*β* = 0.34, 95% CI: 0.24–0.44, *p* < 0.001), dedication (*β* = 0.36, 95% CI: 0.27–0.45, *p* < 0.001), and vigor (*β* = 0.25, 95% CI: 0.15–0.35, *p* < 0.001).

EI was also positively associated with job performance (*β* = 0.41, 95% CI: 0.31–0.51, *p* < 0.001), explaining 17% of the variance (*R* ^2^ = 0.17). This association was evident for task performance (*β* = 0.38, 95% CI: 0.29–0.47, *p* < 0.001) and contextual performance (*β* = 0.42, 95% CI: 0.33–0.53, *p* < 0.001). In contrast, EI was not significantly associated with CWB (*β* = 0.00, 95% CI: −0.09–0.09, *p* = 0.932) (Table 5 and Figure 3).

**TABLE 5:** Standardized path coefficients (*β*), 95% CIs, *p* values, and *R* ^2^ for the association between emotional intelligence, and organizational commitment, work engagement, and job performance (*N* = 254).

Outcome variable	Standardized path coefficient (*β*) [95% CI)	*p* value	Explained variance
Organizational commitment	0.34 [0.21, 0.47]	< 0.001^∗^	0.12
Normative commitment	0.33 [0.22, 0.44]	< 0.001^∗^	0.11
Continuance commitment	0.26 [0.15, 0.37]	< 0.001^∗^	0.07
Affective commitment	0.20 [0.04, 0.36]	0.013^∗^	0.04
Work engagement	0.37 [0.27, 0.45]	< 0.001^∗^	0.14
Absorption	0.34 [0.24, 0.44]	< 0.001^∗^	0.11
Dedication	0.36 [0.27, 0.45]	< 0.001^∗^	0.13
Vigor	0.25 [0.15, 0.35]	< 0.001^∗^	0.06
Job performance	0.41 [0.31, 0.51]	< 0.001^∗^	0.17
Task performance	0.38 [0.29, 0.47]	< 0.001^∗^	0.15
Contextual performance	0.42 [0.33, 0.53]	< 0.001^∗^	0.17
Counterproductive work behaviors	0.00 [−0.09, 0.09]	0.932	0.00

*Note:* ^∗^Statistically significant at *p* < 0.05.

**FIGURE 3:**
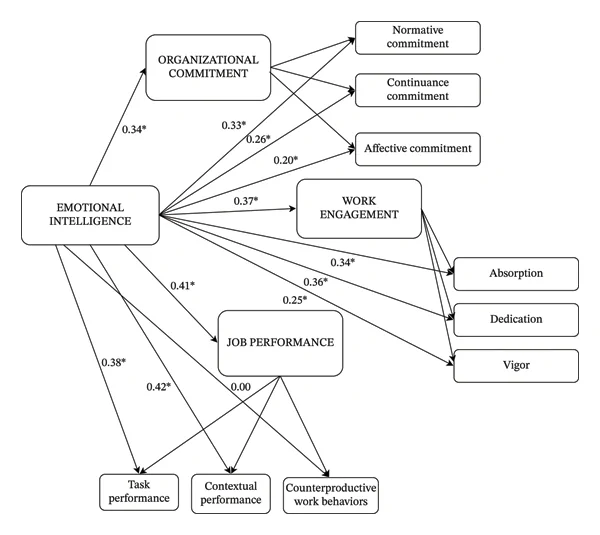
Path diagram illustrating the association between emotional intelligence and organizational commitment, work engagement, and job performance among midwives (*N* = 254).

## 6. Discussion

This study assessed the association between self‐reported EI and organizational commitment, work engagement, and job performance among midwives in the Tamale Metropolis. The findings indicate that EI was significantly associated with these work‐related outcomes. These findings add to the existing literature by providing evidence from midwives working in a resource constrained, LMIC setting. However, given the confirmatory and cross‐sectional design of the study, the findings should be interpreted as evidence of association rather than causation. The observed relationships may also reflect the influence of other workplace and individual factors, including workload intensity, staffing adequacy, leadership climate, remuneration, burnout, and organizational support, which were not fully accounted for in the present analysis.

The findings have potential implications for midwifery management and workforce development. However, they should not be interpreted as demonstrating that increasing EI would necessarily improve organizational commitment, work engagement, or job performance. Rather, the findings suggest that EI may be a relevant factor to consider alongside broader organizational and structural conditions when examining midwives’ workplace outcomes. Further longitudinal and intervention‐based research is needed to determine whether changes in EI are associated with subsequent changes in these outcomes and to clarify the extent to which organizational factors influence these relationships.

Although the observed path coefficients (*β* = 0.34–0.41) are conventionally interpreted as moderate effects, their clinical and managerial significance should be considered cautiously. In applied workforce terms, a *β* of this magnitude suggests that targeted EI interventions could produce modest but meaningful shifts in commitment, engagement, and performance scores, rather than transformative change. Given the resource constraints characteristic of the Tamale Metropolis health system, even modest, low‐cost improvements in these outcomes may be practically valuable; however, stakeholders should not expect EI training alone to substantially offset the effects of structural deficits such as understaffing or inadequate remuneration.

The study found that only 13.4% of midwives had high EI. This implies that the majority of midwives in the Tamale Metropolis may lack the emotional skills required to effectively manage their own emotions and those of others in high‐stress healthcare environments. This is particularly concerning given that midwives frequently encounter emotionally charged situations such as obstetric emergencies, maternal loss, patient anxiety, and interpersonal conflicts [53, 54]. From a practical perspective, this finding suggests that midwifery managers cannot assume that emotional competencies develop naturally through clinical exposure alone. On a practical level, managers may need to deliberately integrate EI into routine professional development activities, such as short in‐service training sessions, reflective practice meetings, or continuous professional development (CPD) programs that focus on emotional awareness, stress management, and interpersonal communication. However, this result differs from a past study by Alipour et al. [55], where more than half (61.2%) of midwives had a high level of EI, and from Zolfaghary et al. [56], where midwives’ EI was moderate. These discrepancies may reflect contextual differences in healthcare systems, workload intensity, staffing levels, leadership practices, and access to psychosocial support. Importantly, the low EI levels observed in this study should not be interpreted as individual deficiencies but rather as indicators of limited institutional investment in emotional skills development. The study, therefore, recommends that healthcare organizations in the Tamale Metropolis prioritize EI training as part of professional development programs for midwives, given their low EI levels. Therefore, healthcare managers and nursing/midwifery educators should consider integrating cost‐effective EI‐enhancing activities, such as quarterly reflective sessions, peer‐learning discussions, and brief competency‐based workshops, into existing professional development structures rather than relying solely on individual efforts to develop emotional competencies.

The study further observed that age influenced the EI of midwives, with EI significantly increasing with age. This may be attributed to the accumulation of life and professional experiences that enhance emotional regulation, empathy, and coping skills over time. Older midwives may have developed greater emotional resilience through prolonged exposure to challenging clinical situations. This finding aligns with studies by Snowden et al. [57], Chen et al. [58], and Lartey et al. [59]. From a managerial standpoint, this suggests that older midwives could be intentionally engaged as mentors or peer supporters, helping younger colleagues navigate emotionally demanding situations and develop adaptive coping strategies. Such mentorship programs could be structured informally, for example, through regular reflective sessions or case discussions, making them practical within resource‐limited settings. However, it is also possible that less emotionally resilient midwives exit the profession earlier due to burnout or job dissatisfaction, which may partially explain the higher EI observed among older midwives. It is, therefore, recommended that healthcare institutions institutionalize structured mentorship and peer‐support systems that pair younger midwives with more experienced colleagues to facilitate emotional learning, resilience building, and professional socialization.

The study found a moderate positive association between EI and overall organizational commitment (*β* = 0.34), suggesting that EI has a meaningful relationship with midwives’ commitment to their organizations, although it represents only one of several factors influencing organizational attachment. This finding indicates that midwives with higher EI may be more likely to develop stronger loyalty and identification with their organizations, which can contribute to lower turnover intentions [60, 61] and higher job satisfaction [21, 62]. This result is in accordance with studies by ElDin and Abd El Rahman [22], Arage et al. [23], and Lingyu et al. [24], which report that higher EI positively influences organizational commitment. Moreover, EI was associated with all dimensions of organizational commitment—normative commitment (feeling obligated to stay), continuance commitment (perceived costs of leaving), and affective commitment (emotional attachment to the organization)—and showed agreement with a previous study [63]. This indicates that emotionally intelligent midwives are more likely to remain loyal to their organizations, even in challenging circumstances. Practically, this suggests that fostering EI may serve as a nonfinancial retention strategy, particularly relevant in settings where financial incentives are limited. However, organizational commitment is also strongly shaped by leadership practices, fairness in promotion, workload distribution, and remuneration, which may moderate or amplify the observed influence of EI.

The study demonstrated a moderate positive association between EI and overall work engagement (*β* = 0.37), indicating that EI has a meaningful contribution to midwives’ psychological involvement and investment in their work. This suggests that midwives with higher EI may be better able to remain focused, motivated, and committed despite demanding clinical environments. This is consistent with prior studies by Chikobvu and Harunavamwe [25], Pérez‐Fuentes et al. [29], Turjuman and Alilyyani [31], and Bartlett [30], which demonstrated positive relationships between EI and work engagement. Additionally, all dimensions of work engagement, including absorption, dedication, and vigor, were positively associated with EI. This suggests that EI‐enhancing interventions may contribute to improved workplace engagement. However, given that EI explained only 14% of the variance in work engagement, interventions should also address other important determinants of engagement, including workload balance, staffing adequacy, supportive supervision, and opportunities for professional growth.

The study identified a moderate positive association between EI and overall job performance (*β* = 0.41), representing the strongest association observed among the main outcomes and suggesting that EI has practical relevance for supporting effective professional performance among midwives. This finding implies that emotionally intelligent midwives may be better equipped to regulate emotions, manage interpersonal demands, and maintain effective clinical functioning. This was similarly observed in studies by Agustin et al. [38], Hashmi et al. [36], and Cheraghi et al. [37], which also identified EI as having a positive association with job performance and its dimensions, task performance, and contextual performance. The positive relationship suggests that emotionally intelligent midwives may be better equipped to manage workplace demands, maintain professional relationships, and effectively perform their clinical responsibilities.

Interestingly, the study found no significant relationship between EI and CWBs. This finding should be interpreted cautiously because the estimated effect size was negligible (*β* = 0.00), and the sample may have had limited statistical power to detect a very small association between EI and CWB. Additionally, because CWB represents socially undesirable workplace behaviors, self‐report measurement may have resulted in underreporting and restricted variability, reducing the likelihood of identifying significant associations. Alternatively, counterproductive behaviors may be influenced more strongly by organizational and environmental conditions, such as excessive workload, inadequate staffing, poor leadership climate, and burnout, rather than individual emotional competencies alone. Therefore, strategies aimed at reducing counterproductive behaviors should combine individual‐focused interventions, such as EI development, with organizational approaches that improve workplace conditions.

Overall, although EI was significantly associated with the assessed work outcomes, the relatively low explained variance observed in the models (*R* ^2^ = 0.12–0.17) indicates that EI accounts for only a limited proportion of variation in organizational commitment, work engagement, and job performance. The fact that 83%–88% of the variance in these outcomes remains unexplained is a critical finding in its own right and should not be treated as a peripheral caveat. It indicates that organizational commitment, work engagement, and job performance among midwives are shaped predominantly by factors outside individual emotional competence, most plausibly organizational and structural variables such as staffing ratios, supervisory support, remuneration adequacy, workload distribution, and professional autonomy. This reinforces the need for future research to adopt multilevel models that simultaneously capture individual (EI) and organizational‐level predictors, rather than attributing workforce outcomes primarily to individual psychological traits. Therefore, EI should be considered an important but complementary factor within a broader workforce development framework rather than a standalone determinant of midwives’ professional outcomes.

## 7. Limitations

Despite its contributions, this study has several limitations that should be considered when interpreting the findings. First, the data were obtained from midwives working in only three hospitals within the Tamale Metropolis, which may limit the generalizability of the results to other settings or regions. Second, the reliance on self‐reported questionnaires introduces the possibility of response bias, as participants may have overestimated or underestimated their EI, work engagement, job performance, or commitment levels. This reliance on self‐reported measures may also have resulted in an overestimation of the observed associations due to social desirability and common method effects, meaning that the reported effect sizes may not fully reflect the magnitude of these relationships in the broader population.

Third, the study involved multiple statistical comparisons, particularly in the analyses of the subdimensions of EI and their associations with organizational commitment, work engagement, and job performance. We have acknowledged in the Limitations section that conducting multiple comparisons increases the probability of obtaining statistically significant findings by chance and therefore increases the risk of Type I error. Although the primary analyses were guided by prespecified outcomes, the subscale findings should be interpreted cautiously and considered supportive rather than definitive evidence of specific dimension level associations.

Fourth, the cross‐sectional study design precludes causal inferences regarding the observed relationships among EI and work outcomes. Future research could address these limitations by employing longitudinal or quasiexperimental designs to clarify causal pathways and to disentangle the independent effects of EI from organizational and environmental factors. In addition, the effectiveness of EI interventions could be evaluated prospectively using pre‐ and postintervention assessments of EI, organizational commitment, work engagement, and job performance, complemented by qualitative feedback from midwives.

## 8. Implications for Nursing Practice

Given the generally low levels of EI observed, feasible workforce development strategies are warranted. At the individual level, hospital administrators, nursing and midwifery managers, and professional bodies could integrate EI training into existing CPD programs through quarterly reflective practice sessions, peer‐support groups, mentorship, and brief skills–based workshops, potentially strengthening emotional regulation, interpersonal skills, and professional functioning among midwives. At the organizational level, nursing and midwifery managers could establish structured mentorship programs, supportive supervision, and staff recognition systems, while incorporating relevant emotional competencies into staff appraisal and professional development frameworks, potentially improving organizational commitment, work engagement, and job performance.

Training institutions could also integrate EI competencies into undergraduate and professional curricula, particularly providing greater developmental support for younger midwives, to promote early development of emotional competencies and better preparation for professional practice. Implementation should involve relevant managers, educators, institutional leaders, and professional bodies and should consider potential organizational resistance to training or changes in established practices, thereby supporting sustainable implementation and acceptance of such initiatives. Collectively, these individual‐ and organizational‐level strategies could contribute to improved staff well‐being, professional relationships, quality of patient care, and overall workplace functioning, while complementing broader efforts to improve staffing conditions and leadership support.

Furthermore, future studies should use longitudinal and intervention designs to determine whether improvements in EI are associated with subsequent changes in organizational commitment, work engagement, and job performance. Multisite studies across different regions of Ghana are needed to assess the consistency and generalizability of these findings. Further research should also examine specific EI competencies, identify organizational barriers and facilitators to implementation, and evaluate the feasibility and effectiveness of different EI development approaches among midwives.

## 9. Conclusions

This is the first study of its kind conducted in Tamale Metropolis, Ghana, to assess the association between self‐reported EI and organizational commitment, work engagement, and job performance among midwives. This study found that EI among midwives in the Tamale Metropolis was generally low, with only a small proportion demonstrating high EI. After adjustment, older age was associated with higher EI. EI was also positively associated with organizational commitment, work engagement, and job performance, including normative, continuance, and affective commitment; absorption, dedication, and vigor; and task and contextual performance. Given the cross‐sectional design and single‐metropolis setting, these findings should be interpreted as associations and not assumed to be causal or broadly generalizable. Nevertheless, the findings suggest that EI may be relevant to midwives’ professional functioning and warrant attention at both individual and organizational levels. From a nursing ethics perspective, strengthening emotional competencies may also support professional responsibility, respectful patient care, and constructive nurse–patient relationships [64].

## Author Contributions

Letitia Chanayireh: conceptualization, data curation, investigation, methodology, project administration, supervision, and validation. Zariatu Yakubu: conceptualization, data curation, project administration, and writing–original draft. Sheila Kansig Puotege: conceptualization, data curation, project administration, and writing–original draft. Beatrice Asakiya: conceptualization, investigation, and writing–original draft and review and editing. Mary Tusungu: conceptualization, investigation, and writing–original draft and review and editing. Mudasir Mohammed Ibrahim: conceptualization, data curation, formal analysis, methodology, validation, visualization, and writing–original draft and review and editing. Iddrisu Mohammed Sisala: conceptualization, data curation, funding acquisition, investigation, project administration, supervision, and writing–review and editing. Abubakari Wuni: conceptualization, methodology, project administration, and validation.

## Funding

No funding was received for the conduct or publication of this research.

## Disclosure

All authors have read and approved the final version of the manuscript.

## Conflicts of Interest

The authors declare no conflicts of interest.

## Supporting Information

Additional supporting information can be found online in the Supporting Information section.

## Supporting information


**Supporting Information** Supporting File 1 is an R Markdown document presenting the statistical analysis and output underlying Table 5. It includes the path analysis results for the associations between emotional intelligence and organizational commitment, work engagement, and job performance.

## Data Availability

The data that support the findings of this study are available from the corresponding author upon reasonable request.
