# Supplementary material for: Association Between Emotional Intelligence and Work Outcomes Among Midwives in Northern Ghana: A Path Analysis Study
Source: J Nurs Manag. 2026 Oct 7;2026:6200182. doi: 10.1155/jonm/6200182 (PMC13646483; doi:10.1155/jonm/6200182)
Supplement: Supplementary file 1 [file JONM-2026-6200182-s001.pdf]

Analysis with MLR

Chanayireh et al

2026-01-19

```
#Set working directory
setwd("/Users/XXXXXXX/XXXXXXX/XXXXXXX/XXXXXXX
XXXXXXX/XXXXXXX")

#Load packages
library(tidyverse)

## ── Attaching core tidyverse packages ──────────────────────── tidyverse
2.0.0 ──
## ✔ dplyr     1.1.4     ✔ readr     2.1.4
## ✔ forcats   1.0.0     ✔ stringr   1.5.1
## ✔ ggplot2   4.0.0     ✔ tibble    3.2.1
## ✔ lubridate 1.9.4     ✔ tidyr     1.3.0
## ✔ purrr     1.0.2
## ── Conflicts ──────────────────────────────────────────
tidyverse_conflicts() ──
## ✖ dplyr::filter() masks stats::filter()
## ✖ dplyr::lag()    masks stats::lag()
## ℹ Use the conflicted package (<http://conflicted.r-lib.org/>) to force
all conflicts to become errors

library(lavaan)

## This is lavaan 0.6-20
## lavaan is FREE software! Please report any bugs.

library(semPlot)
library(readxl)

#Import data
mydata <- read_excel("/Users/XXXXXXX/XXXXXXX/XXXXXXX
/XXXXXXX/XXXXXXX/Excel data import.xlsx")

attach(mydata)

#Path analysis
model1 <- "O_C~E_I
          W_E ~ E_I
          W_P~E_I

"
fitmodel1 <- sem(model1, data=mydata, estimator="MLR")
```

```r
#Check fit statistics measures
summary(fitmodel1, fit.measures=TRUE, standardized=TRUE)
```

```
## lavaan 0.6-20 ended normally after 36 iterations
##
##   Estimator                                         ML
##   Optimization method                           NLMINB
##   Number of model parameters                         9
##
##   Number of observations                           254
##
## Model Test User Model:
##                                               Standard      Scaled
##   Test Statistic                                 0.000       0.000
##   Degrees of freedom                                 0           0
##
## Model Test Baseline Model:
##
##   Test statistic                               230.051     171.227
##   Degrees of freedom                                 6           6
##   P-value                                        0.000       0.000
##   Scaling correction factor                                  1.344
##
## User Model versus Baseline Model:
##
##   Comparative Fit Index (CFI)                    1.000       1.000
##   Tucker-Lewis Index (TLI)                       1.000       1.000
##
##   Robust Comparative Fit Index (CFI)                            NA
##   Robust Tucker-Lewis Index (TLI)                               NA
##
## Loglikelihood and Information Criteria:
##
##   Loglikelihood user model (H0)               -698.194    -698.194
##   Loglikelihood unrestricted model (H1)       -698.194    -698.194
##
##   Akaike (AIC)                                1414.389    1414.389
##   Bayesian (BIC)                              1446.225    1446.225
##   Sample-size adjusted Bayesian (SABIC)       1417.693    1417.693
##
## Root Mean Square Error of Approximation:
##
##   RMSEA                                          0.000          NA
##   90 Percent confidence interval - lower         0.000          NA
##   90 Percent confidence interval - upper         0.000          NA
##   P-value H_0: RMSEA <= 0.050                       NA          NA
##   P-value H_0: RMSEA >= 0.080                       NA          NA
##
##   Robust RMSEA                                              0.000
##   90 Percent confidence interval - lower                    0.000
```

```
##   90 Percent confidence interval - upper                     0.000
##   P-value H_0: Robust RMSEA <= 0.050                            NA
##   P-value H_0: Robust RMSEA >= 0.080                            NA
##
## Standardized Root Mean Square Residual:
##
##   SRMR                                          0.000       0.000
##
## Parameter Estimates:
##
##   Standard errors                            Sandwich
##   Information bread                          Observed
##   Observed information based on               Hessian
##
## Regressions:
##                    Estimate  Std.Err  z-value  P(>|z|)   Std.lv  Std.all
##   O_C ~
##     E_I               0.012    0.002    5.263    0.000    0.012    0.339
##   W_E ~
##     E_I               0.024    0.003    7.599    0.000    0.024    0.368
##   W_P ~
##     E_I               0.018    0.002    8.352    0.000    0.018    0.413
##
## Covariances:
##                    Estimate  Std.Err  z-value  P(>|z|)   Std.lv  Std.all
##  .O_C ~~
##    .W_E              -0.000    0.030   -0.003    0.997   -0.000   -0.000
##    .W_P               0.009    0.021    0.457    0.648    0.009    0.030
##  .W_E ~~
##    .W_P               0.328    0.041    8.095    0.000    0.328    0.602
##
## Variances:
##                    Estimate  Std.Err  z-value  P(>|z|)   Std.lv  Std.all
##    .O_C               0.258    0.031    8.331    0.000    0.258    0.885
##    .W_E               0.798    0.068   11.807    0.000    0.798    0.865
##    .W_P               0.373    0.033   11.235    0.000    0.373    0.830
```
